# Functional Characterization of a Strong Bi-directional Constitutive Plant Promoter Isolated from *Cotton Leaf Curl Burewala Virus*

**DOI:** 10.1371/journal.pone.0121656

**Published:** 2015-03-23

**Authors:** Zainul A. Khan, Malik Z. Abdin, Jawaid A. Khan

**Affiliations:** 1 Plant Virus Laboratory, Department of Biosciences, Jamia Millia Islamia (Central University), New Delhi, India; 2 Department of Biotechnology, Hamdard University, New Delhi, India; Aligarh Muslim University, INDIA

## Abstract

*Cotton leaf curl Burewala virus* (CLCuBuV), belonging to the genus *Begomovirus*, possesses single-stranded monopartite DNA genome. The bidirectional promoters representing *Rep* and coat protein (*CP*) genes of CLCuBuV were characterized and their efficacy was assayed. Rep and CP promoters of CLCuBuV and 35S promoter of *Cauliflower mosaic virus* (CaMV) were fused with β-*glucuronidase* (*GUS*) and green fluorescent protein (*GFP*) reporter genes. GUS activity in individual plant cells driven by Rep, CP and 35S promoters was estimated using real-time PCR and fluorometric GUS assay. Histochemical staining of GUS in transformed tobacco (*Nicotiana tabacum* cv. Xanthi) leaves showed highest expression driven by Rep promoter followed by 35S promoter and CP promoter. The expression level of *GUS* driven by Rep promoter in transformed tobacco plants was shown to be two to four-fold higher than that of 35S promoter, while the expression by CP promoter was slightly lower. Further, the expression of *GFP* was monitored in agroinfiltrated leaves of *N*. *benthamiana*, *N*. *tabacum* and cotton (*Gossypium hirsutum*) plants using confocal laser scanning microscopy. Rep promoter showed strong consistent transient expression in tobacco and cotton leaves as compared to 35S promoter. The strong constitutive CLCuBuV Rep promoter developed in this study could be very useful for high level expression of transgenes in a wide variety of plant cells.

## Introduction

Members of the family *Geminiviridae*, commonly called as geminiviruses, are characterized by having single-stranded, circular DNA genome of 2.5–3.0 kb, which is either monopartite or bipartite and encapsidated within virions having geminate morphology. Different geminiviruses can infect either monocotyledonous or dicotyledonous plants, and collectively, these viruses cause a variety of major crop diseases. On the basis of genome organization, insect vector and host range, the family, *Geminiviridae* is differentiated into seven genera, *Curtovirus*, *Topocuvirus*, *Mastrivirus*, *Begomovirus*, *Becurtovirus*, *Eragrovirus* and *Turncurtovirus* [1–3]. *Begomovirus* is the largest genus among geminiviruses having more than 200 species and transmitted by whitefly (*Bemisia tabaci*) vector. Monopartite begomoviruses have one genomic component (DNA-A) with six open reading frames (ORFs). The coat protein gene (*AV1*) and *AV2* are expressed from the viral sense strand while *Rep* gene (*AC1*), *AC2*, *AC3* and *AC4* are expressed from the complimentary sense strand [4]. Most monopartite begomoviruses are associated with satellite molecule called betasatellite which is often required for the development of wild type symptom in naturally-infected host plant. It requires a helper begomovirus for its replication, systemic infection and whitefly vector-mediated transmission [5–8]. Some begomovirus betasatellite disease complexes are associated with a nanovirus like component named alphasatellite [8–11].

The large intergenic region (LIR) of monopartite begomovirus and common region (CR) of bipartite begomovirus contain bidirectional promoter which includes *cis*-acting regulatory elements, transcription factor binding sites (TFBs) and transcription start site required for the control of gene expression and replication [12]. Functional analysis of CR of bipartite and LIR of several monopartite geminiviruses have demonstrated that this region, located between 5′ ends of the first complimentary and virion sense ORFs, possesses promoter activity and is essential for bidirectional transcription of both complimentary as well as virion sense genes [13–16]. In the absence of transcriptional activator protein AC2, complimentary sense (Rep) promoter of *African cassava mosaic virus* (ACMV), *Tomato golden mosaic virus* (TGMV) and *Cotton leaf curl Multan virus* (CLCuMuV) showed much stronger activity than the virion sense (CP) promoter [17–19]. LIR sequence of several geminiviruses share little sequence homology to one another but there are several conserved motifs present in almost all geminiviruses, like, two TATA boxes located at either side of LIR and GC-rich box (except ACMV) [20]. The TATA box motif and G-box motif located 15 nucleotide (nt) upstream of the stem-loop motif in TGMV is essential for activation of complementary sense gene transcription [21]. In *Maize streak virus*, deletion of GC-rich region resulted in 70% reduction of promoter activity [22]. The LIR also contains the origin of replication of viral genome. The iterated elements (8–13 nt) and stem-loop motif have been identified in the region of the putative TATA box of the complimentary sense promoter [20,22,23].

*Cotton leaf curl Burewala virus* (CLCuBuV) is a whitefly-transmitted monopartite begomovirus and has recombinant genome composed of sequences derived from CLCuMuV and *Cotton leaf curl Kokhran virus* (CLCuKoV) that were associated with cotton leaf curl disease (CLCuD) during 1990s [24–26]. The genome of monopartite begomovirus is organized into six ORFs viz. *AC1* (Rep), *AC2* (TrAp), *AC3* (REn), *AC4*, *AV1* (CP) and *AV2* which are transcribed bidirectionally from the LIR [15]. Rep is involved in viral replication and acts as transcriptional repressor of its own expression by binding to the iterated elements located in the LIR [12,27–29].

In the present study, the bidirectional promoters of *Rep* and *CP* genes from the LIR sequence of CLCuBuV were isolated and characterized. Further, expression patterns and activities of *GUS* reporter gene tagged with Rep and CP promoters were monitored, quantified and compared to CaMV 35S promoter in the transformed tobacco plants. Simultaneously, the *GFP* reporter gene tagged with Rep and CP promoters was also monitored both in tobacco and cotton leaves by agroinfiltration method. The observations of the present study revealed a promising future of CLCuBuV Rep promoter as a potential tool for ectopic gene expression in plants. Further, it may be an alternate for the most widely used CaMV 35S promoter in plant biotechnology applications.

## Materials and Methods

### Cloning and sequencing of CLCuBuV genome

**Fig 1:**
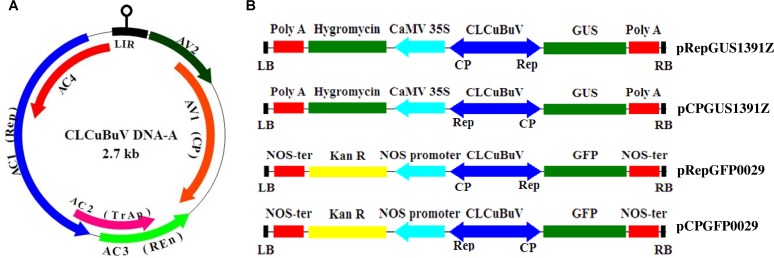
Genome organization and Schematic representation of different promoter constructs. (A) Genome organization of DNA-A component of *Cotton leaf curl Burewala virus*. 6 ORFs are shown as arrows *AV1* (CP) and *AV2* on virion sense strand and *AC1* (Rep), *AC2* (TrAp), *AC3* (REn) and *AC4* on complimentary sense strand. The large intergenic region (LIR), containing the potential stem-loop structure with the nonanucleotide TAATATTAC conserved among all geminiviruses. (B) Schematic representation of the expression vectors showing the cloning of Rep and CP promoters. (CP: Coat protein; Rep: Replication initiation protein; TrAP: Transcription activator protein; REn: Replication enhancer protein; CaMV 35S: *Cauliflower mosaic virus* 35S promoter; CLCuBuV: *Cotton leaf curl Burewala virus*; LB: Left-border sequence of T-DNA; RB: Right-border sequence of T-DNA).

Cotton plants (*Gossypium hirsutum*) showing typical leaf curling, vein thickening and enation on the abaxial side of leaf were collected from a field in Lucknow, U. P., India (26° 50′ 49.2″ N, 80° 56′ 49.2″ E). The leaf sample of the diseased cotton plant was collected from a private land with the verbal consent of the owner to pick the leaf sample for academic purpose (*Gossypium hirsutum* is not an endangered or protected species), in September of 2010. Total genomic DNA from diseased cotton leaf was isolated using DNAeasy plant mini kit (QIAGEN, Germany) following the manufacturer’s instructions. Begomoviral complex was confirmed as described by Kumar *et al*. [30]. Full-length genome of CLCuBuV was amplified using templiPhi DNA amplification kit (GE healthcare, USA) as per manufacturer’s instructions. The RCA (rolling circle amplification) product was partially digested with 1 U of *Hind*III restriction enzyme (Fermentas, USA) and incubated at 37°C for 25 min. The purified DNA fragment was ligated into pBluscriptSK+ vector and transformed into *Escherichia coli* DH5α cells. The clone was sequenced by automated sequencer (Ocimum Biosolutions Ltd., India) and the nucleotide sequence was submitted in GenBank under the accession number KF767352. Genome organization of DNA-A component of CLCuBuV is represented in Fig. 1A.

### Cloning of large intergenic region

Primers viz. PR For and PR Rev were designed on the basis of the submitted nucleotide sequence (KF767352) for amplification of LIR (Table 1). The amplification was performed in 50 μl of reaction mixture containing 100 ng of template DNA (RCA product), 50 pmol of each primer, 1x *Taq* DNA polymerase buffer, 200 μM dNTPs and 1.25 U AmpliTaq DNA Polymerase (Applied Biosystems, USA). The reactions were conducted at 94°C for 5 min as initial denaturation followed by 30 cycles of 94°C for 1 min, 48°C for 2 min, 72°C for 1 min and final extension at 72°C for 5 min. The amplified DNA product was run on 1.5% agarose gel, stained with EtBr and visualized in Gel documentation system (BIO-RAD, USA). The PCR product of the expected size *ca*. 350 bp was recovered from the agarose gel and purified by using QIAEXII Gel Extraction Kit (QIAGEN, Germany). The quantity and purity of gel eluted DNA were measured by BioPhotometer plus (Eppendorf, Germany) and cloned into pDrive PCR cloning vector (QIAGEN, Germany). The positive colonies were confirmed by PCR and restriction digestion, and nucleotide sequences were determined using an automated sequencer (ABI3730XL, Applied Biosystems, USA).

**Table 1:** List of oligonucleotide primers used for amplifying promoter fragments and genes.

Name of primer	Primer sequences in 5′–3′
PR	5′AGATCTCATTGACTTTGGTCAATTAGAG 3′ (For)
	5′AGATCTCATACACCTAAAACCGTGAACGG 3′ (Rev)
LIR	5′TCTAGATTGGGGGGAGCCATTGACT 3′ (For)
	5′TCTAGATTCCTAGCCCTTATTACCAG 3′ (Rev)
2G	5′GTATCAGTGTGCATGGCTGGATATG 3′ (For)
	5′GCGAGTGAAGATCCCTTTCTTGTTA 3′ (Rev)
Actin	5′GCATAGAGGGAAAGCACAGC 3′ (For)
	5′TGGTAGGTATGGGCCAGAAA 3′ (Rev)

For: forward primer; Rev: reverse primer

### Sequence analysis

The *cis*-regulatory elements of the bidirectional promoter sequence of CLCuBuV were analyzed using the Plant Cis-Acting Regulatory Element (Plant CARE) database [31] and Cister: Cis-Element Cluster Finder [32]. Transcription Factor Binding sites (TFBs) were identified by using Plant cis-acting regulatory DNA elements (PLACE) database and PlantPAN: Plant Promoter Analysis Navigator [33,34]. Neural Network Promoter Prediction (NNPP) server was used for the prediction of transcription start site (TSS) [35].

### Development of LIR-GUS and LIR-GFP gene fusion vectors

Four plant expression vectors viz. pRepGUS1391Z, pCPGUS1391Z, pRepGFP0029 and pCPGFP0029 representing bidirectional CLCuBuV Rep/Cp promoter were constructed (Fig. 1B). For generating pRepGUS1391Z and pCPGUS1391Z vectors, LIR of CLCuBuV, previously cloned in pDrive vector, was digested with *Bgl*II restriction enzyme (Fermentas, USA) and ligated into pCAMBIA1391Z vector (Promoterless Vector kindly provided by Dr Sunil K Mukherjee, ICGEB, New Delhi, India). The integration and orientation of the bidirectional promoter was confirmed by PCR amplification and restriction digestion. The resulting plasmid was named pRepGUS1391Z and pCPGUS1391Z. For constructing LIR-GFP fusion constructs, LIR was first amplified using primer pair LIR For and LIR Rev (Table 1). The PCR amplicon was cloned in pGEM-T Easy Vector (Promega, USA). GFP cloned in pBI121 vector was digested with *Eco*RI and *Bam*HI restriction enzymes (Fermentas, USA) and ligated into plant expression vector pGreen0029 (kindly provided by Dr Neera Bhalla Sarin, Jawaharlal Nehru University, New Delhi, India). The resulting plasmid was named pGFP0029. LIR previously cloned into pGEM-T Easy Vector was digested with *Xba*I restriction enzyme (Fermentas, USA) and ligated into pGFP0029 vector. The orientation of the bidirectional promoter of CLCuBuV was confirmed by PCR, restriction digestion and sequencing. The resulting gene constructs were named as pRepGFP0029 and pCPGFP0029. pCAMBIA2301 containing CaMV 35S promoter-GUS-NOS polyA cassette and pBI121 containing CaMV 35S promoter-GFP-T NOS cassette were used as positive control.

### Plant transformation

The plant expression vectors were introduced into *Agrobacterium tumefaciens* strain LBA4404 by freeze thaw method [36]. The gene constructs, namely pRepGFP0029 and pCPGFP0029, were mobilized in *A*. *tumefaciens* strain LBA4404 along with pSoup helper plasmid [37]. *Agrobacterium*-mediated transformation of leaf discs of *Nicotiana tabacum* cv. Xanthi was carried out essentially as described by Horsch *et al*. [38]. Regenerated hygromycin-resistant plants were grown in glasshouse and the presence of the promoter was confirmed by PCR.

### Histochemical localization of GUS activity

GUS staining was performed essentially as described [39]. Apical leaves of transformed tobacco plants (or tissue samples), one month after sub culture were incubated overnight at 37°C in a solution containing 1 mM X-Gluc (Fermentas, USA), 50 mM sodium phosphate buffer pH 7.0, 0.5 mM potassium ferricyanide, 0.5 mM potassium ferrocyanide, 1 mM Na_2_EDTA, 0.1% Triton X100. The chlorophyll was removed using 70% ethanol. Petioles and leaves were sectioned by hand after staining. The samples were observed and photographed under Nikon 80I microscope (Nikon, Japan).

### Fluorometric GUS activity assay

Quantitative GUS fluorometric assays were conducted according to the method of Jafferson [40] by measuring the fluorescence of 4-methylumbelliferone (MU) formed as a result of the cleavage of 4-methylumbelliferyl-β-D-glucuronide (MUG, Himedia) by GUS with a spectrofluorometer FP-6300 (JASCO, Japan). GUS activity was calculated as the production of MU from MUG in pmol/min/μg of protein.

### RT-PCR analysis of GUS transcripts driven by CLCuBuV Rep, CLCuBuV CP and CaMV 35S promoters

Total RNA was isolated from leaves of transformed *Nicotiana tabacum* cv. Xanthi using RNeasy Plant Mini kit (QIAGEN, Germany) according to manufacturer’s instructions. The cDNA for RT-PCR was synthesized by reverse transcription of 5 μg of total RNA using Long Range 2 Step RT-PCR Kit (QIAGEN, Germany) following manufacturer’s instructions. The gene specific primers 2G For and 2G Rev, and reference gene primers Actin For and Actin Rev were designed with Primer3 software [41]. PCR was performed by 2G For and 2G Rev and Actin For and Actin Rev primers (Table 1). The PCR was conducted at 95°C for 5 min as initial denaturation followed by 35 cycles of 95°C for 30 s, 54°C for 30 s, 72°C for 1 min and final extension at 72°C for 5 min. The amplified PCR products were separated on 1.5% agarose gel, stained with EtBr and visualized in gel documentation system (BIO-RAD, USA).

### Reverse transcription quantitative real-time PCR (RT-qPCR)

RT-qPCR was performed using a LightCycler ^R^96 Real-Time PCR System (Roche, USA) using 2G For and 2G Rev, Actin For and Actin Rev primers. RT-qPCR was carried out in 25 μl reaction volumes consisting of 100 ng cDNA, 0.5 μM forward and reverse primers and 1x Maxima SYBR Green Master Mix (Thermo Scientific, USA). PCR conditions included an initial denaturation at 95°C for 10 min, followed by a 45 cycle 3 step amplification with denaturation at 95°C for 30 s, annealing at 54°C for 30 s and extension at 72°C for 1 min. Duplex of all samples were run, a negative control and non reverse transcription control of the master mix was included with all qPCR experiments. The expression level of the *GUS* gene was normalized using the actin gene as an endogenous control. The relative quantification of samples was computed using LightCycler 96 Software version 1.1.0.1320. Relative fluorescence was detected by the Cq values.

### Transient expression of GFP

A single colony of *A*. *tumefaciens* (strain LBA4404) carrying individual gene constructs (pRepGFP0029, pCPGFP0029 and pBIGFP121) was inoculated in 10 ml of Yeast Extract Mannitol (YEM) medium supplemented with kanamycin (50 μg/ml) and rifampicin (50 μg/ml). It was grown at 28°C for 48 h while shaking at 200 rpm. 500 μl of primary culture of *A*. *tumefaciens* suspension was then inoculated into 50 ml of YEM medium containing 10 mM MES (pH 5.6), 100 μM acetosyringone and antibiotics (50 μg/ml). It was grown at 28°C for 16 h while shaking at 200 rpm. The *Agrobacterium* cells were harvested by centrifuging at 3,000 rpm for 15 min and resuspended in MMMA medium (MS salts, 10 mM MES pH 5.6, 10 mM MgCl_2_ and 100μM acetosyringone) and incubated at room temperature for 2 h before agroinfiltration.

Agroinfiltration experiments were performed as essentially described previously [42]. Seeds of *N*. *benthamiana*, *N*. *tabacum* and *G*. *hirsutum* were planted and grown in growth chamber at 25°C under 16 h: 8 h, light: dark photoperiod. Plants were grown for 4 to 6 weeks before infiltration. The bacterial suspension was infiltrated into the intercellular space on the abaxial side of each intact leaf by using 2 ml syringe without needle. The plants were then placed in growth chamber (25°C, photoperiod, 16 h: 8 h, light: dark). The expression of *GFP* was monitored 72 h post infiltration under UV light. Further, the *GFP* expression was monitored under fluorescence microscope ECLIPSE 80i (Nikon, Japan) in the wavelength 330–380 nm.

### Promoter expression analysis (CLSM)

Leaves of tobacco and cotton were agroinfiltrated with the individual promoter constructs carrying *GFP* reporter gene for assaying their transient activities as described earlier. The fluorescent images were captured with CLSM (Leica Microsystems, Germany) using LAS AF (Leica Application Suit Advanced Fluorescence) software under oil-immersion objective (40.0X1.15OIL). To excite the expressed *GFP* in leaves the AOTF of 488 nm (at 40%) was used and the fluorescence emissions were collected between 501 and 598 nm as described earlier [43].

## Results

### Sequence analysis of CLCuBuV promoter

**Fig 2:**
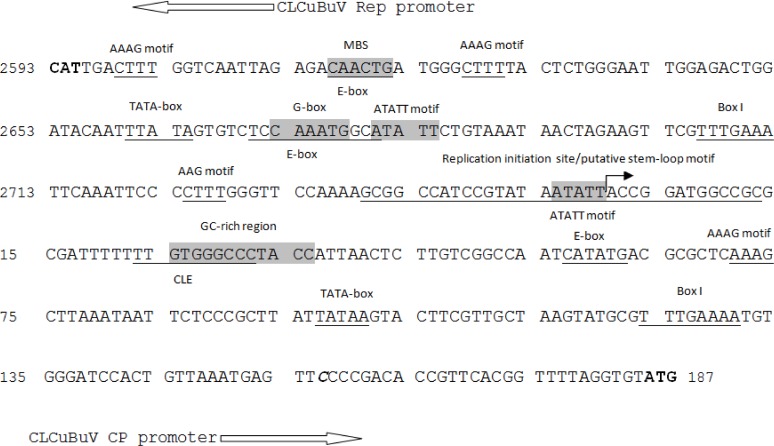
The nucleotide sequence of the large intergenic region of *Cotton leaf curl Burewala virus* determined in this study. Putative *cis*-acting elements including TATA box, GC-rich sequence, stem-loop motif, E box, conserved late element (CLE), AAAG motif are indicated. The putative translation start sites ATG and CAT are indicated in bold, and the transcription start site is indicated in bold italics.

The bidirectional promoter was isolated from the LIR monopartite genome (DNA-A) of CLCuBuV. Its size was 353 nucleotides long (Fig. 2). Analysis of the promoter sequence of CLCuBuV revealed the presence of several putative *cis* elements such as G-box, stem-loop motif, TATA boxes, a GC-rich region (GTGGGCCCTACC) and a conserved late element (CLE) (Fig. 2, Tables 2 and S1). The TATA boxes and the GC rich region have been shown to be essential for the activity of the geminivirus promoters. The putative CLE (TTGTGGGCCCTA) sequence at +189 was suggested to be a potential functional target for *AC2* gene to trans-activate the virion sense gene transcription [20,44]. The promoter sequence also revealed several potential TFBs, the E-box motifs (CANNTG) at sites +24, +80, +223, −29, −85 and −228 [45], the maize Dof transcription factor recognition core sequence AAAG at sites −7, −35, −131 and +237 [46] and the root specific ATATT motifs at sites +88, +162 and −165 [47] (Table 3). Other potential TFBs and *cis* regulatory DNA elements were also identified by searching the Plant CARE, PLACE and PlantPAN databases as listed in Tables 2, 3 and S2.

**Table 2:** List of putative *cis*-acting regulatory elements present in the CLCuBuV promoter region (Plant CARE).

S. No.	TFBs	Function	Organism	Sequence	Matrix score	Strand	Position
1	Box I	light responsive element	*Pisum sativum*	TTTCAAA	7	−	114
				TTTCAAA	7	−	290
2	Box-W1	fungal elicitor responsive element	*Petroselinum crispum*	TTGACC	6	−	11
3	C-repeat/DRE	regulatory element involved in cold- and dehydration-responsiveness	*Arabidopsis thaliana*	TGGCCGAC	8	−	213
4	CAAT-box	common *cis*-acting element in promoter and enhancer regions	*Hordeum vulgare*	CAAT	4	−	2
			*Brassica rapa*	CAAAT	5	+	123
			*Glycine max*	CAATT	5	+	64
			*H*. *vulgare*	CAAT	4	+	219
			*G*. *max*	CAATT	5	−	48
			*A*. *thaliana*	CCAAT	5	+	218
			*B*. *rapa*	CAAAT	5	+	80
			*G*. *max*	CAATT	5	+	14
			*A*. *thaliana*	CCAAT	5	−	49
5	CGTCA-motif	*cis*-acting regulatory element involved in the MeJA-responsiveness	*H*. *vulgare*	CGTCA	5	−	227
6	ERE	ethylene-responsive element	*Dianthus caryophyllus*	ATTTCAAA	8	−	114
7	G-Box	*cis*-acting regulatory element involved in light responsiveness	*Triticum aestivum*	TCCACATGGCA	10	+	78
8	GC-motif	Unknown	*Oryza sativa*	GCCGCGCG	8	+	175
9	MBS	MYB binding site involved in drought-inducibility	*A*. *thaliana*	CAACTG	6	+	24
10	Skn-1_motif	*cis*-acting regulatory element required for endosperm expression	*O*. *sativa*	GTCAT	5	−	226
11	TATA-box	core promoter element around -30 of transcription start	*Lycopersicon esculentum*	TTTTA	5	+	36
			*G*. *max*	TAATA	5	+	160
			*A*. *thaliana*	TATAA	5	−	68
			*A*. *thaliana*	TATAA	5	−	262
			*G*. *max*	TAATA	5	−	260
			*Nicotiana tabacum*	tcTATAAAta	9	+	92
			*L*. *esculentum*	TTTTA	5	+	241
			*Antirrhinum majus*	TATAAATT	8	−	65
			*G*. *max*	TAATA	5	−	163
			*A*. *thaliana*	TATA	4	+	69
			*A*. *thaliana*	TATA	4	+	263
			*A*. *thaliana*	TATAAA	6	−	67
			*B*. *napus*	ATTATA	6	+	261
			*A*. *thaliana*	TATA	4	+	158
12	TGACG-motif	*cis*-acting regulatory element involved in the MeJA-responsiveness	*H*. *vulgare*	TGACG	5	+	227
13	W-box	Unknown	*A*. *thaliana*	TTGACC	6	−	11
14	Circadian	*cis*-acting regulatory element involved in circadian control	*L*. *esculentum*	CAANNNNATC	6	−	182

**Table 3:** List of putative transcripitional factor binding sites (TFBs) present in CLCuBuV promoter region (PLACE database).

S. No.	TFBs	PLACE ID	PLACE accession	Sequence	Copy number	Reference
1	Auxin responsive	ARFAT (ARF = Auxin response factor)	S000270	TGTCTC	2	[48]
2	Cytokinin responsive	ARR1AT	S000454	NGATT	2	[49]
3	Legumin specific	CAATBOX1	S000028	CAAT	5	[50]
4	Mesohpyll Specific	CACTFTPPCA1	S000449	YACT	6	[51]
5	Auxin responsive	CATATGGMSAUR	S000370	CATATG	2	[52]
6	Signal responsive	CGCGBOXAT	S000501	VCGCGB	4	[53]
7	Endosperm specific	DOFCOREZM	S000265	AAAG	5	[54]
8	Abscisic acid response element	EBOXBNNAPA	S000144	CANNTG	6	[55]
9	Abscisic acid signaling	MYCCONSENSUSAT	S000407	CANNTG	6	[56]
10	Root specific	ROOTMOTIFTAPOX1	S000098	ATATT	3	[57]
11	Auxin responsive	SURECOREATSULTR11	S000499	GAGAC	3	[58]
12	Light specific	SORLIP2AT	S000483	GGGCC	2	[59]
13	Wound specific	WBOXATNPR1	S000390	TTGAC	2	[60]
14	Wound specific	WBOXNTERF3	S000457	TGACY	2	[61]
15	Gibberellin specific	WRKY71OS	S000447	TGAC	3	[62]
16	Water stress specific	MYBCORE	S000176	CNGTTR	2	[63]
17	Light responsive	GT1CONSENSUS	S000198	GRWAAW	2	[64]

### Histochemical localization of GUS expression driven by CLCuBuV Rep, CLCuBuV CP and CaMV 35S promoter constructs

**Fig 3:**
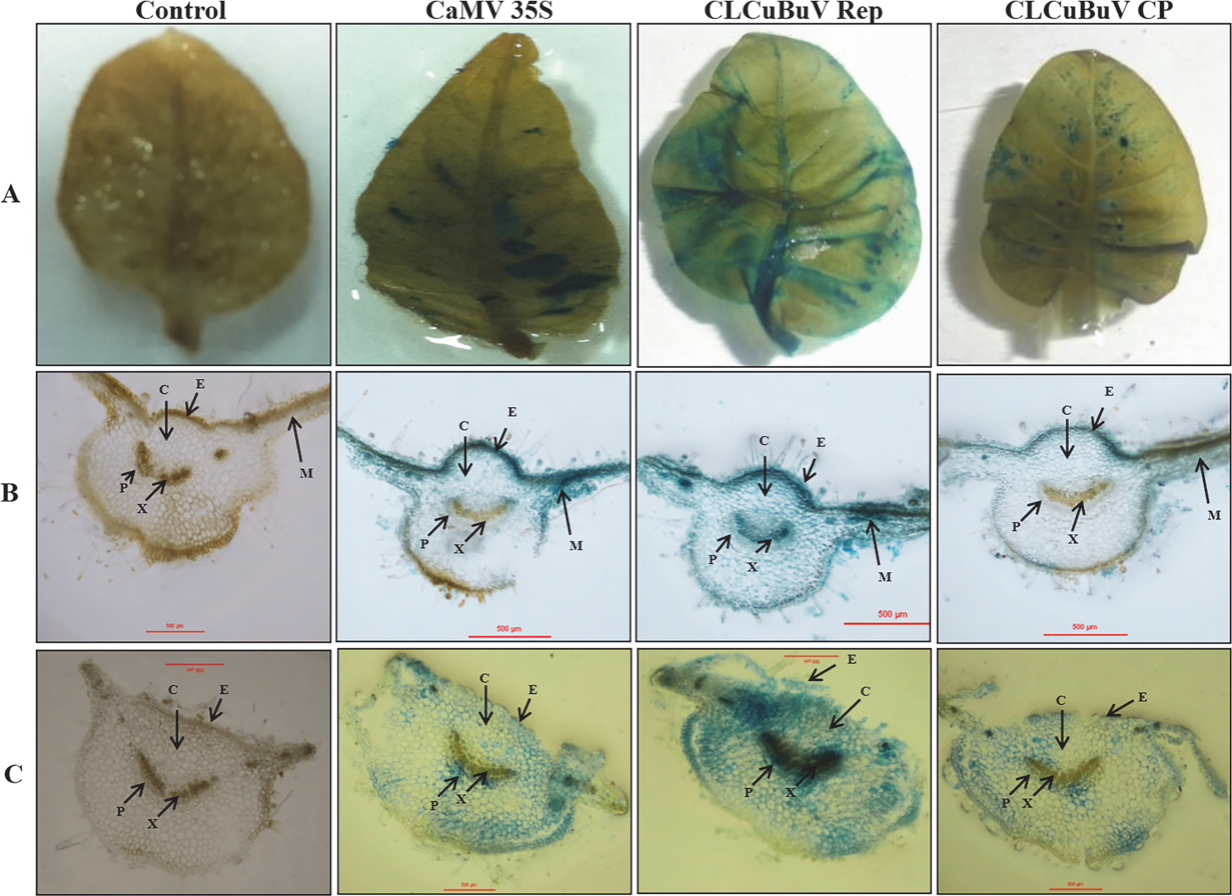
Histochemical localization of GUS activity (blue coloration) directed by *Cauliflower mosaic virus* 35S, *Cotton leaf curl Burewala virus* Rep and *Cotton leaf curl Burewala virus* CP promoters in transformed *Nicotiana tabacum* leaves. (A) Whole leaves, (B) Transverse section through the midrib and leaf lamina showing the presence of GUS signal in mesophyll cells, trichomes and vascular tissues (phloem and xylem parenchyma cells). (C) Transverse section through the petiole. (C: Cortex; E: Epidermis; M: Mesophyll; P: Phloem; X: Xylem).

PCR positive transformed tobacco plants were stained for GUS activity using X-Gluc staining solution. Staining patterns of representative plant leaves are shown in Fig. 3. GUS staining of the transgenic plants containing CLCuBuV Rep was shown to be stronger than that of the plants carrying CaMV 35S promoter and CLCuBuV CP. The expression pattern of CLCuBuV CP promoter was observed to be weaker than that of CaMV 35S promoter (Fig. 3A). The transverse section of leaf (vascular tissue, lamina and midrib) of tobacco plants transformed with CLCuBuV Rep promoter construct, showed strong *GUS* expression in the mesophyll (spongy and palisade) cells and trichome (Fig. 3B and C).

### GUS activity of CLCuBuV Rep and CP promoter in transformed tobacco

**Fig 4:**
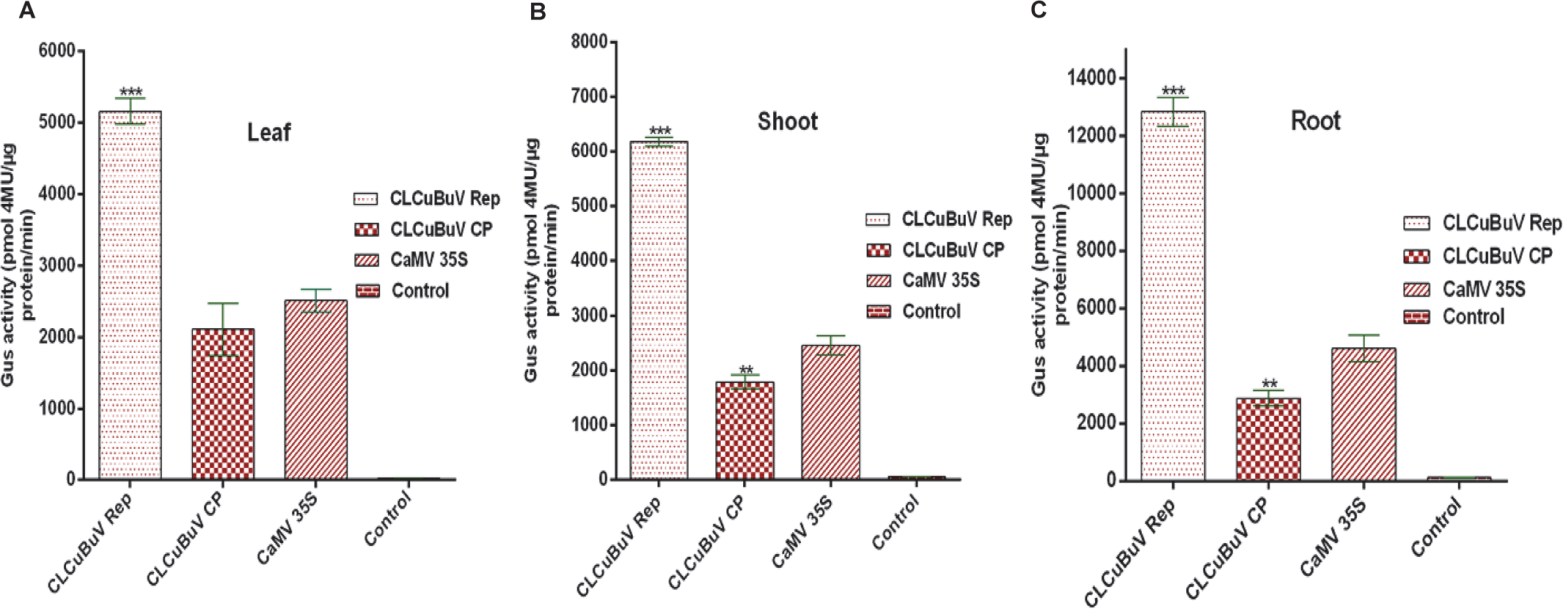
Monitoring of GUS activity in 28 days old transformed *Nicotiana tabacum* plants. Soluble protein extracts from (A) leaves, (B) shoots, and (C) roots were used for GUS assay. The data represent average ± SD (*n* = 3; **, p < 0.01, ***, p < 0.001 when compared with CaMV 35S promoter by student’s *t* test) of each construct: CaMV 35S promoter (pCAMBIA2301), CLCuBuV Rep promoter (pRepGUS1391Z) and CLCuBuV CP promoter (pCPGUS1391Z), and wild type control tobacco. Experiment was repeated three times with several replicates. Error bars indicate SE. (n: Number of samples; p: p values).

The GUS activity in hygromycin-resistant transformed tobacco plants was assayed by a fluorometric quantification method and measured in pmol 4-methyl umbelliferone (4MU)/min/μg proteins. The transgenic tobacco plants containing promoter-GUS constructs were regenerated from hygromycin-resistant calli. Transgenic lines of tobacco plants were established for each construct. Presence of the *GUS* gene and promoter sequence was confirmed by PCR. The GUS activity was assayed in mature leaves, shoots and roots of 4 weeks old transgenic lines for each construct. The average level of GUS activity in mature leaves, transformed with pRepGUS1391Z, pCPGUS1391Z and pCAMBIA2301 gene constructs, were 5175.12 (±180.74), 2106.72 (±363.07) and 2512.42 (±156.65) pmol 4MU/min/μg proteins, respectively (Fig. 4A) (p < 0.001 by Student’s *t* test).

Average value of GUS activity in shoots, transformed with pRepGUS1391Z, pCPGUS1391Z and pCAMBIA2301 constructs, were 6179.65 (±85.99), 1788.34 (±126.22) and 2452.95 (±174.45) pmol 4MU/min/μg proteins, respectively (Fig. 4B) (*p* < 0.001 by student’s *t* test).

Average value of GUS activity in roots, transformed with pRepGUS1391Z, pCPGUS1391Z and pCAMBIA2301 constructs, were 12835.02 (±492.25), 2882.52 (±262.83) and 4607.38 (±466.80) pmol 4MU/min/μg proteins, respectively (Fig. 4C) (*p* < 0.001 by student’s *t* test). The control tobacco plants showed no expression of *GUS*.

The efficacy of CLCuBuV Rep promoter was found to be higher than CaMV 35S promoter in leaves, shoots and roots of all transgenic plants. However, the expression level of CLCuBuV CP promoter was lower than that of CaMV 35S promoter in the tissues of leaves, shoots and roots of the transformed plants. The GUS activity in the roots appeared to be higher than that of the shoots and leaves (Fig. 4A, B and C).

### Transcriptional regulation of GUS

**Fig 5:**
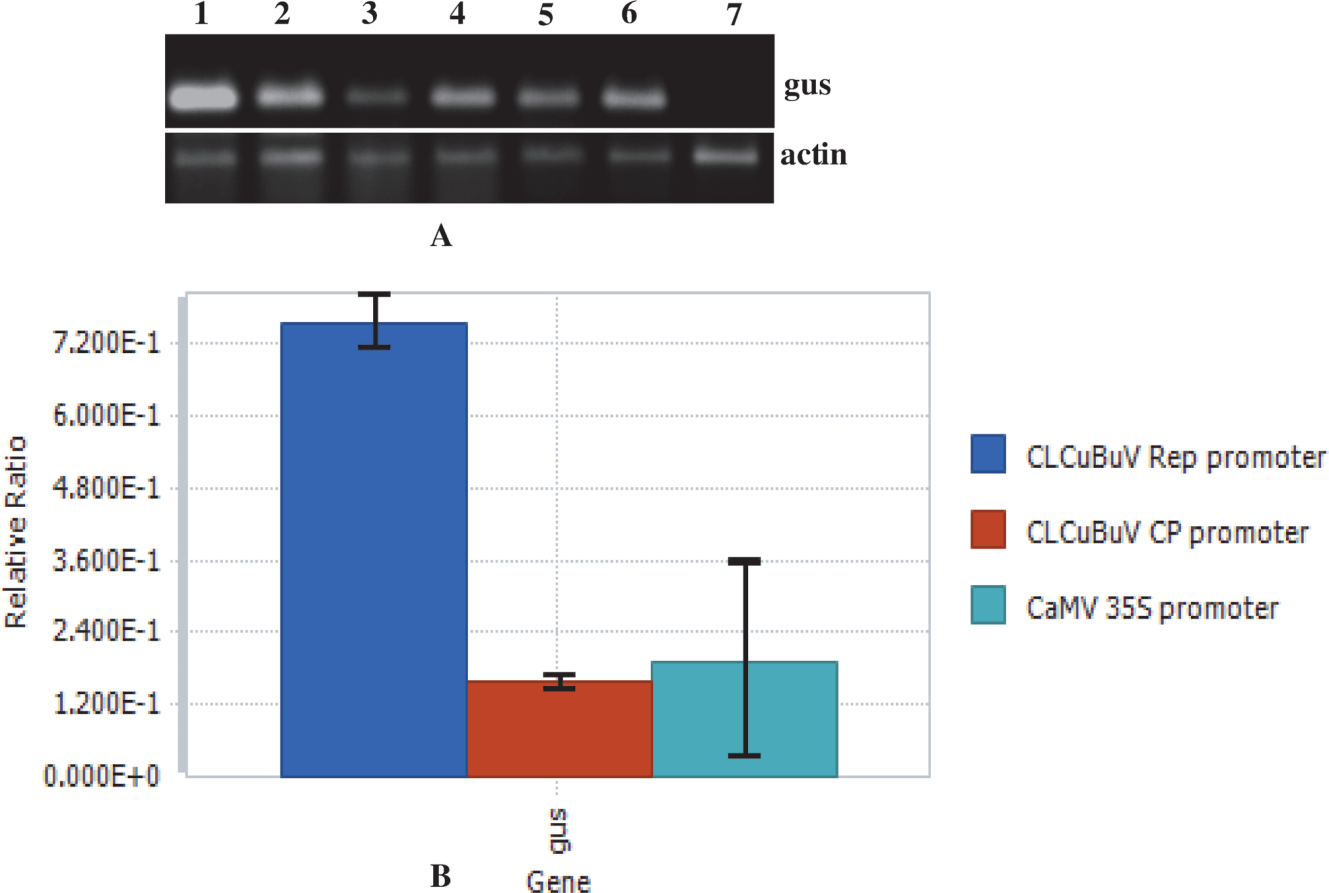
Expression analysis of GUS transcripts under the control of *Cotton leaf curl Burewala virus* promoters in transformed *Nicotiana tabacum* plants. (A) RT-PCR amplification of GUS transcripts from total RNA isolated from *N*. *tabacum* plants transformed by CLCuBuV Rep (1 and 2), CLCuBuV CP (3 and 4) and CaMV 35S (5 and 6) promoter constructs, wild type control tobacco plant (7). Electrophoresis of RT-PCR amplification of actin transcripts isolated from total RNA from transformed *N*. *tabacum* plants generated from the same constructs. (B) The relative ratio of GUS transcripts quantified by Reverse transcription quantitative real-time PCR. All the experiments were repeated three times with several replicates. Error bars indicate SE.

To confirm the expression of *GUS* gene at mRNA level, RT-PCR analysis was performed using total RNA samples from transgenic and non-transgenic plants of *Nicotiana tabacum* cv. Xanthi. The RT-PCR analysis of transgenic plants showed that the transgenes were expressed differentially in these transgenic plants (Fig. 5A). Further, no amplification was observed in non-transgenic tobacco plant employing GUS-specific primers but amplification with actin-specific primers was observed in both transgenic and non-transgenic tobacco plants (Fig. 5A). The CLCuBuV Rep promoter transformed plants had shown higher expression level than those of CLCuBuV CP and CaMV 35S promoter. The qRT-PCR was performed to study the expression of *GUS* gene under the control of CLCuBuV Rep, CLCuBuV CP and CaMV 35S promoter. Results revealed that *GUS* gene transcript level was four-fold higher in CLCuBuV Rep promoter transformed plants compared to that of CaMV 35S promoter (Fig. 5B). Transcript level of *GUS* gene under the control of CaMV 35S promoter was observed slightly higher than that of CLCuBuV CP promoter.

### Visualization of transiently expressed GFP in tobacco and cotton leaves

**Fig 6:**
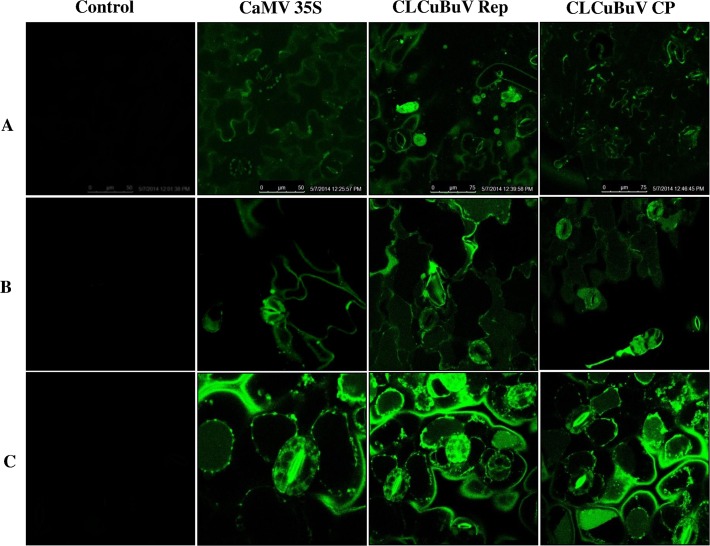
Visualization of green fluorescent protein expression by a confocal laser scanning microscope. (A) *Nicotiana benthamiana*, (B) *N*. *tabacum*, and (C) *Gossypium hirsutum* leaves agroinfiltrated with *Cauliflower mosaic virus* 35S promoter (pBIGFP121), *Cotton leaf curl Burewala virus* Rep promoter (pRepGFP0029) and *Cotton leaf curl Burewala virus* CP promoter (pCPGFP0029) constructs.

**Fig 7:**
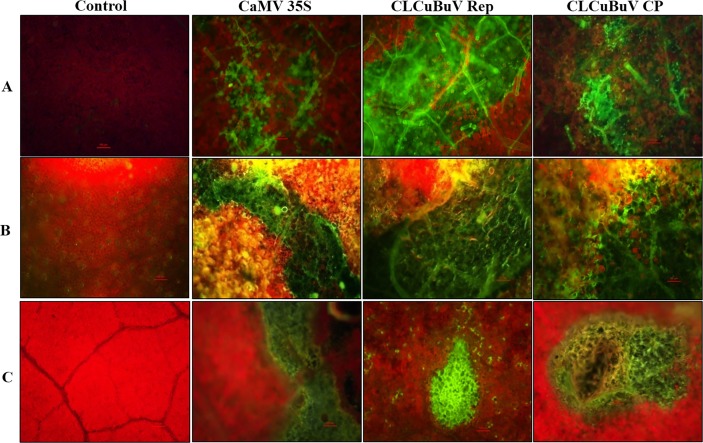
Visualization of green fluorescent protein expression under fluorescent microscope (Nikon 80I). (A) *Nicotiana benthamiana*, (B) *N*. *tabacum*, and (C) *Gossypium hirsutum* leaves agroinfiltrated with *Cauliflower mosaic virus* 35S promoter (pBIGFP121), *Cotton leaf curl Burewala virus* Rep promoter (pRepGFP0029) and *Cotton leaf curl Burewala virus* CP promoter (pCPGFP0029) constructs.

The expression of *GFP* was detected in leaves of agroinfiltrated plants of both tobacco and cotton under ultraviolet light 3 days post-infiltration. Fluorescent image of leaves expressing *GFP* in tobacco and cotton were captured by the confocal microscope and fluorescence microscope as described earlier and are presented in Figs. 6 and 7, respectively. Higher fluorescent intensity of GFP was found in both tobacco and cotton leaves agroinfiltrated with CLCuBuV Rep promoter construct as compared to CaMV 35S and CLCuBuV CP promoter constructs.

## Discussion

Efficient genetic modification of plants for agronomic traits requires the use of efficient regulatory sequences to drive the expression of trans-genes in specific plant tissues or at certain developmental stages. The pattern of expression provided by different promoters must be analyzed before they can be used to generate transgenic plants commercially. In this study the sequence of LIR from CLCuBuV was analyzed. We have compared the expression patterns of *GUS* gene driven by CLCuBuV Rep and CLCuBuV CP promoters with CaMV35S promoter in transformed transgenic tobacco plants. The highest expression of *GUS* gene was obtained using the CLCuBuV Rep promoter followed by the CaMV 35S promoter and the lowest activity was observed with CLCuBuV CP promoter (Figs. 4 and 5). In the transgenic tobacco plants the CLCuBuV Rep promoter showed strong GUS activity while the CLCuBuV CP promoter showed very weak activity. This may be due to the absence of AC2 gene product in CLCuBuV [24–26]. Role of transactivator AC2 protein for activation of virion sense promoter has already been reported [65,66]. The supremacy of Rep (AC1) promoter over the CP (AV1) promoter, in the absence of transactivtor AC2 protein was demonstrated in CLCuMuV [19], ACMV [18], *Wheat dwarf virus* [67] and *Mungbean yellow mosaic India virus* [68]. Gene expression process in geminivirus shows an early expression of complimentary sense gene, whose products are then involved in viral replication such as AC1 (Rep) or they may act as transcription activator for the virion sense gene expression, such as AC2 (TrAp). In contrast the expression of virion sense genes usually appears later and requires complimentary sense gene product/products for activation. The staining of GUS activity showed that the CLCuBuV promoter was active in all the tobacco cells observed. The strongest histochemical staining for GUS activity was observed in the mesophyll cells and vascular tissue of the leaf transformed with CLCuBuV Rep promoter construct (Fig. 3). The quantitative analysis of GUS assay clearly revealed that CLCuBuV Rep promoter had two to four-fold higher activity than the CaMV 35S promoter, while CLCuBuV CP promoter was lagging behind CLCuBuV Rep and CaMV 35S promoter, demonstrating least activity among the three (Figs. 4 and 5).

Transcription level of the *GUS* reporter gene driven by CLCuBuV was quantified for the first time in this study. RT-PCR and RT-qPCR based analysis of GUS-specific transcripts isolated from transgenic tobacco plants containing CLCuBuV Rep, CLCuBuV CP and CaMV 35S promoter constructs clearly showed a fair correlation between the steady-state level of mRNA (transcript level) and GUS activity (protein level) (Fig. 5). Based on the above experimental evidence, it was confirmed that the activity of CLCuBuV Rep promoter was significantly higher than that of CaMV 35S and CLCuBuV CP promoters.

For the first time transient expression of *GFP* reporter gene under the control of CLCuBuV promoters in different plants, such as, *N*. *tabacum*, *N*. *benthamiana* and *G*. *hirsutum* was also investigated. Simultaneously, a positive control constitutive promoter CaMV 35S associated with GFP was also used to study the degree of effectiveness between the promoters such as CLCuBuV Rep, CLCuBuV CP and CaMV 35S. In this study, the expression and localization of the *GFP* reporter gene in agroinfiltrated leaves was monitored by confocal laser scanning microscopy (Fig. 6). Our histochemical results clearly showed that expression pattern of *GFP* under the control of CLCuBuV Rep promoter were found to be more effective than CaMV 35S promoter, used in consequent experiments.

Though several groups have carried out similar studies in tobacco and cotton plants, they did not quantify the expression level driven by these promoters at transcriptional level [15,16,19]. Ashraf *et al*. [15] quantified the transiently expressed GUS activity driven by CLCuBuV C1 gene promoter by fluorometric assay. Evidence was provided exhibiting 2–3 fold higher GUS activity of CLCuBuV promoter than that of 35S. In present study, GUS activity was quantified in stably transformed transgenic tobacco plants by RT-qPCR as well as fluorometric assay. RT-qPCR clearly demonstrated that the expression of GUS activity was up to four fold higher than 35S promoter. Further, for the first time, expression of *GFP* reporter gene under the control of CLCuBuV promoters (Rep and CP) was *in situ* localized in three different plant species such as *N*. *tabacum*, *N*. *benthamiana* and *G*. *hirsutum* using confocal laser scanning microscopy. Not only the novelty and degree of effectiveness of these promoters in three different plants (*N*. *tabacum*, *N*. *benthamiana* and *G*. *hirsutum*) was demonstrated, but it also reflects that it may be used to develop other valuable agronomic traits. The observations of the present study reveal a promising future of CLCuBuV Rep promoter as a potential tool for ectopic gene expression in plants, particularly cotton.

## Supporting Information

S1 Table Putative functional *cis*-elements of CLCuBuV promoter (Cister: Cis-element Cluster Finder). (PDF)

S2 Table Transcription factor binding sites in CLCuBuV promoter sequence. (PDF)
