# Supplementary material for: Functional Characterization of a Strong Bi-directional Constitutive Plant Promoter Isolated from Cotton Leaf Curl Burewala Virus
Source: PLoS One. 2015 Mar 23;10(3):e0121656. doi: 10.1371/journal.pone.0121656 (PMC4370823; doi:10.1371/journal.pone.0121656)
Supplement: S1 Table — (PDF) [file pone.0121656.s001.pdf]

S1 Table. Putative functional *cis*-elements of CLCuBuV promoter (Cister: Cis-element Cluster Finder).

S. No.	Type	Position	Strand	Sequence	Probability
1	CCAAT	213 to 228	+	gtcggccaatcatatg	1
2	TATA	59 to 73	-	ggatacaatttatag	0.91
3	TATA	253 to 267	-	tcccgcttattataa	0.74
4	TATA	157 to 171	+	gtataatattaccgg	0.64
5	CCAAT	24 to 39	-	caactgatgggctttt	0.52
6	TATA	289 to 303	+	gtttgaaaatgtggg	0.43
7	CCAAT	43 to 58	-	ctgggaattggagact	0.42
8	TATA	93 to 107	+	ctgtaaataactaga	0.39
9	TATA	235 to 249	-	Tcaaagcttaaataa	0.34
10	TATA	180 to 194	-	Gcgatttttttgtgg	0.24
11	TATA	95 to 109	+	gtaaataactagaag	0.16
12	TATA	178 to 192	-	gcgcgatttttttgt	0.14
13	CCAAT	127 to 142	-	ttccccctttgggttcc	0.13
14	TATA	177 to 191	-	cgcgcgatttttttg	0.11
