# Supplementary material for: Functional Characterization of a Strong Bi-directional Constitutive Plant Promoter Isolated from Cotton Leaf Curl Burewala Virus
Source: PLoS One. 2015 Mar 23;10(3):e0121656. doi: 10.1371/journal.pone.0121656 (PMC4370823; doi:10.1371/journal.pone.0121656)
Supplement: S2 Table — (PDF) [file pone.0121656.s002.pdf]

S2 Table. Transcription factor binding sites in CLCuBuV promoter Sequence.

Transcription factor	Site	Strand	Sequence	Species	Source
AG	75	+	gtctCCAAAtggcatatt	Arabidopsis	TRANSFAC
AG	124	-	aaattccccTTTGGgttc	Arabidopsis	TRANSFAC
AG	137	+	ggttCCAAAagcggccat	Arabidopsis	TRANSFAC
ANT	317	+	tgagttcCCCGAca	Arabidopsis	TRANSFAC
Athb-1	155	-	ccgtATAATattac	Arabidopsis	TRANSFAC
Athb-1	242	-	ttaaATAATtctcc	Arabidopsis	TRANSFAC
Athb-1	256	+	cgcttATTATaagt	Arabidopsis	TRANSFAC
ATHB-5	97	-	aAATAActa	Arabidopsis	TRANSFAC
ATHB-5	244	-	aAATAAttc	Arabidopsis	TRANSFAC
ATHB-5	256	+	cgcTTATTa	Arabidopsis	TRANSFAC
ATHB-9	214	-	tcggccaATCATatgacgc	Arabidopsis	TRANSFAC
Dof2	32	-	ggGCTTTtact	Maize	TRANSFAC
Dof2	140	+	tccaAAAGCgg	Maize	TRANSFAC
Dof2	233	+	gctcAAAGCtt	Maize	TRANSFAC
Dof3	32	-	ggGCTTTtact	Maize	TRANSFAC
Dof3	140	+	tccaAAAGCgg	Maize	TRANSFAC
Dof3	233	+	gctcAAAGCtt	Maize	TRANSFAC
O2	304	-	atccactgttaAATGA	Maize	TRANSFAC
PBF	32	-	gggCTTTTact	Maize	TRANSFAC
PBF	140	+	tccAAAAGcgg	Maize	TRANSFAC
P	196	+	ccCTACCat	Maize	TRANSFAC
MYB.Ph3	332	+	cgttcaCGGTTtt	Petunia	TRANSFAC
MYB.Ph3	99	-	atAACTAgaagtt	Petunia	TRANSFAC
bZIP910	225	+	taTGACGcgctc	Snapdragon	TRANSFAC
bZIP910	221	+	atcataTGACGc	Snapdragon	TRANSFAC
bZIP911	225	+	taTGACGcgctc	Snapdragon	TRANSFAC
bZIP911	225	+	taTGACGcgctc	Snapdragon	TRANSFAC
-10PEHVPSBD	89	+	TATTCT	Barley	PLACE
ABI4	143	-	AAAAGCGG	Maize	JASPER
ARFAT	20	-	GAGACA	Arabidopsis/Soybean/rice	PLACE
ARFAT	20	-	GAGACA	Arabidopsis/Soybean/rice	PLACE
ARFAT	20	-	GAGACA	Arabidopsis/Soybean/rice	PLACE
ARFAT	74	+	TGTCTC	Arabidopsis/Soybean/rice	PLACE
ARFAT	74	+	TGTCTC	Arabidopsis/Soybean/rice	PLACE
ARFAT	74	+	TGTCTC	Arabidopsis/Soybean/rice	PLACE
ARF	20	-	GAGACA	Arabidopsis	AGRIS
ARF	74	+	TGTCTC	Arabidopsis	AGRIS
ARR1AT	181	+	CGATT	Arabidopsis	PLACE
ARR1AT	220	-	AATCA	Arabidopsis	PLACE
ASF1MOTIFCAMV	227	+	TGACG	tobacco/Arabidopsis	PLACE
ASF1MOTIFCAMV	227	+	TGACG	tobacco/Arabidopsis	PLACE
Agamous	141	+	CCAAAAGCGGC	Arabidopsis	JASPER
Agamous	76	-	TCTCCAAATGG	Arabidopsis	JASPER
C8GCARGAT	64	+	CAATTTATAG	Arabidopsis	PLACE
C8GCARGAT	64	-	CAATTTATAG	Arabidopsis	PLACE
CAATBOX1	14	+	CAAT	pea	PLACE

CAATBOX1	2	-	ATTG	pea	PLACE
CAATBOX1	219	+	CAAT	pea	PLACE
CAATBOX1	49	-	ATTG	pea	PLACE
CAATBOX1	64	+	CAAT	pea	PLACE
CARGCW8GAT	64	+	CAATTTATAG	Arabidopsis	PLACE
CARGCW8GAT	64	-	CAATTTATAG	Arabidopsis	PLACE
CATATGGMSAUR	223	+	CATATG	soybean	PLACE
CATATGGMSAUR	223	-	CATATG	soybean	PLACE
CBF1	213	-	GTCGGCCA	Arabidopsis	AGRIS
CBFHV	213	-	GTCGGC	barley	PLACE
CCAATBOX1	218	+	CCAAT	Soybean	PLACE
CCAATBOX1	49	-	ATTGG	Soybean	PLACE
CGCGBOXAT	176	+	CCGCGC	Arabidopsis	PLACE
CGCGBOXAT	176	-	CCGCGC	Arabidopsis	PLACE
CGCGBOXAT	229	+	ACGCGC	Arabidopsis	PLACE
CGCGBOXAT	229	-	ACGCGC	Arabidopsis	PLACE
CIACADIANLELHC	182	-	GATTTTTTTG	tomato	PLACE
Core	16	+	ATTA	Arabidopsis	AGRIS
Core	160	-	TAAT	Arabidopsis	AGRIS
Core	164	+	ATTA	Arabidopsis	AGRIS
Core	203	+	ATTA	Arabidopsis	AGRIS
Core	247	-	TAAT	Arabidopsis	AGRIS
Core	261	+	ATTA	Arabidopsis	AGRIS
DOFCOREZM	132	-	CTTT	maize	PLACE
DOFCOREZM	144	+	AAAG	maize	PLACE
DOFCOREZM	237	+	AAAG	maize	PLACE
DOFCOREZM	35	-	CTTT	maize	PLACE
DOFCOREZM	7	-	CTTT	maize	PLACE
DRECRTCOREAT	213	-	GTCGGC	rice/maize/sunflower	PLACE
DRECRTCOREAT	213	-	GTCGGC	rice/maize/sunflower	PLACE
Dof2	144	+	AAAGCG	Maize	JASPER
Dof2	237	+	AAAGCT	Maize	JASPER
Dof2	33	-	GGCTTT	Maize	JASPER
Dof3	144	+	AAAGCG	Maize	JASPER
Dof3	33	-	GGCTTT	Maize	JASPER
Dof3	5	-	GACTTT	Maize	JASPER
E2FCONSENSUS	215	-	CGGCCAAT	Arabidopsis/tobacco/rice	PLACE
E2FCONSENSUS	215	-	CGGCCAAT	Arabidopsis/tobacco/rice	PLACE
E2FCONSENSUS	215	-	CGGCCAAT	Arabidopsis/tobacco/rice	PLACE
EBOXBNNAPA	223	+	CATATG	rape	PLACE
EBOXBNNAPA	223	-	CATATG	rape	PLACE
EBOXBNNAPA	24	+	CAACTG	rape	PLACE
EBOXBNNAPA	24	-	CAACTG	rape	PLACE
EBOXBNNAPA	80	+	CAAATG	rape	PLACE
EBOXBNNAPA	80	-	CAAATG	rape	PLACE
ELRECOREPCRP1	11	-	GGTCAA	parsley/tobacco	PLACE
ERELEE4	114	-	TTTGAAAT	tomato/carnation/Lycopersicon	PLACE
ERELEE4	119	+	AATTCAAA	tomato/carnation/Lycopersicon	PLACE
GATABOX	60	+	GATA	petunia/Arabidopsis/rice	PLACE

GATABOX	60	+	GATA	petunia/Arabidopsis/rice	PLACE
GATABOX	60	+	GATA	petunia/Arabidopsis/rice	PLACE
GBF5	316	-	ATGAGT	Arabidopsis	AGRIS
GT1CONSENSUS	164	-	ATTACC	pea/oat/rice/tobacco/Arabidopsis	PLACE
GT1CONSENSUS	164	-	ATTACC	pea/oat/rice/tobacco/Arabidopsis	PLACE
GT1CONSENSUS	164	-	ATTACC	pea/oat/rice/tobacco/Arabidopsis	PLACE
GT1CONSENSUS	164	-	ATTACC	pea/oat/rice/tobacco/Arabidopsis	PLACE
GT1CONSENSUS	293	+	GAAAAT	pea/oat/rice/tobacco/Arabidopsis	PLACE
GT1CONSENSUS	293	+	GAAAAT	pea/oat/rice/tobacco/Arabidopsis	PLACE
GT1CONSENSUS	293	+	GAAAAT	pea/oat/rice/tobacco/Arabidopsis	PLACE
GT1CONSENSUS	293	+	GAAAAT	pea/oat/rice/tobacco/Arabidopsis	PLACE
HBOXCONSENSUSPVCHS	197	+	CCTACCATTAACTCT	bean/tobacco	PLACE
HBOXCONSENSUSPVCHS	197	+	CCTACCATTAACTCT	bean/tobacco	PLACE
HMG-1	35	+	CTTTTACTC	Pea	JASPER
LTRECOREATCOR15	213	-	GTCGG	Arabidopsis/rape	PLACE
LTRECOREATCOR15	213	-	GTCGG	Arabidopsis/rape	PLACE
LTRECOREATCOR15	325	+	CCGAC	Arabidopsis/rape	PLACE
LTRECOREATCOR15	325	+	CCGAC	Arabidopsis/rape	PLACE
MNB1A	144	+	AAAGC	Maize	JASPER
MNB1A	237	+	AAAGC	Maize	JASPER
MNB1A	34	-	GCTTT	Maize	JASPER
MNB1A	6	-	ACTTT	Maize	JASPER
MYB.ph3	108	-	AGTTCGTTT	Petunia	JASPER
MYB2CONSENSUSAT	24	+	CAACTG	Arabidopsis	PLACE
MYBCOREATCYCB1	331	-	CCGTT	Arabidopsis	PLACE
MYBCORE	24	-	CAACTG	Arabidopsis/petunia	PLACE
MYBCORE	24	-	CAACTG	Arabidopsis/petunia	PLACE
MYBCORE	309	+	CTGTTA	Arabidopsis/petunia	PLACE
MYBCORE	309	+	CTGTTA	Arabidopsis/petunia	PLACE
MYBPZM	197	+	CCTACC	maize	PLACE
MYBST1	59	+	GGATA	potato	PLACE
MYCCONSENSUSAT	223	+	CATATG	Arabidopsis	PLACE
MYCCONSENSUSAT	223	-	CATATG	Arabidopsis	PLACE
MYCCONSENSUSAT	24	+	CAACTG	Arabidopsis	PLACE
MYCCONSENSUSAT	24	-	CAACTG	Arabidopsis	PLACE
MYCCONSENSUSAT	80	+	CAAATG	Arabidopsis	PLACE
MYCCONSENSUSAT	80	-	CAAATG	Arabidopsis	PLACE
NODCON2GM	208	+	CTCTT	soybean	PLACE
OSE2ROOTNODULE	208	+	CTCTT	bean/Medicago/soybean/Sesbania	PLACE
OSE2ROOTNODULE	208	+	CTCTT	bean/Medicago/soybean/Sesbania	PLACE
OSE2ROOTNODULE	208	+	CTCTT	bean/Medicago/soybean/Sesbania	PLACE
P1BS	86	+	GCATATTC	Arabidopsis/tomato/Medicago/barley	PLACE
P1BS	86	+	GCATATTC	Arabidopsis/tomato/Medicago/barley	PLACE
P1BS	86	+	GCATATTC	Arabidopsis/tomato/Medicago/barley	PLACE
P1BS	86	+	GCATATTC	Arabidopsis/tomato/Medicago/barley	PLACE
P1BS	86	-	GCATATTC	Arabidopsis/tomato/Medicago/barley	PLACE
P1BS	86	-	GCATATTC	Arabidopsis/tomato/Medicago/barley	PLACE
P1BS	86	-	GCATATTC	Arabidopsis/tomato/Medicago/barley	PLACE
P1BS	86	-	GCATATTC	Arabidopsis/tomato/Medicago/barley	PLACE

PBF	144	+	AAAGC	Maize	JASPER
PBF	237	+	AAAGC	Maize	JASPER
PBF	34	-	GCTTT	Maize	JASPER
PBF	6	-	ACTTT	Maize	JASPER
POLASIG3	245	+	AATAAT	maize	PLACE
PREATPRODH	316	-	ATGAGT	Arabidopsis	PLACE
S1FBOXSORPS1L21	199	-	TACCAT	soybean	PLACE
SEBFCONSSTPR10A	20	-	GAGACAA	potato	PLACE
SEF3MOTIFGM	135	-	TGGGTT	soybean	PLACE
SITEIIATCYTC	192	+	TGGGCC	Arabidopsis/rice	PLACE
SITEIIATCYTC	192	+	TGGGCC	Arabidopsis/rice	PLACE
SITEIIATCYTC	31	+	TGGGCT	Arabidopsis/rice	PLACE
SITEIIATCYTC	31	+	TGGGCT	Arabidopsis/rice	PLACE
SORLIP2AT	193	+	GGGCC	Arabidopsis	PLACE
SORLIP2AT	194	-	GGCCC	Arabidopsis	PLACE
SURECOREATSULTR11	20	+	GAGAC	Arabidopsis	PLACE
SURECOREATSULTR11	53	+	GAGAC	Arabidopsis	PLACE
SURECOREATSULTR11	75	-	GTCTC	Arabidopsis	PLACE
TATABOX2	66	-	ATTTATA	pea/tobacco/bean	PLACE
TATABOX2	66	-	ATTTATA	pea/tobacco/bean	PLACE
TATABOX2	66	-	ATTTATA	pea/tobacco/bean	PLACE
TATABOX5	244	-	AAATAA	pea	PLACE
TATABOX5	97	-	AAATAA	pea	PLACE
TATABOXOSPAL	242	-	TTAAATA	rice	PLACE
TATCCAOSAMY	58	-	TGGATA	rice	PLACE
TBOXATGAPB	6	+	ACTTTG	Arabidopsis	PLACE
WBOXATNPR1	12	-	GTCAA	Arabidopsis	PLACE
WBOXATNPR1	3	+	TTGAC	Arabidopsis	PLACE
WBOXHVISO1	4	+	TGACT	barley	PLACE
WBOXNTERF3	11	-	GGTCA	tobacco	PLACE
WBOXNTERF3	4	+	TGACT	tobacco	PLACE
WRKY71OS	12	-	GTCA	rice/parsley	PLACE
WRKY71OS	227	+	TGAC	rice/parsley	PLACE
WRKY71OS	4	+	TGAC	rice/parsley	PLACE
WUSATAg	201	-	CCATTAA	rice	PLACE
